# 2024 Update on Cerebral Embolic Protection After Transcatheter Aortic Valve Replacement

**DOI:** 10.3390/jcm13237256

**Published:** 2024-11-28

**Authors:** Tomasz Gasior

**Affiliations:** Collegium Medicum—Faculty of Medicine, WSB University, 41-300 Dabrowa Gornicza, Poland; tgasior@wsb.edu.pl

**Keywords:** cerebral embolic protection, transcatheter aortic valve replacement (TAVR), transcatheter aortic valve implantation (TAVI), stroke, cerebral embolism

## Abstract

Cerebral embolic protection (CEP) during transcatheter aortic valve replacement (TAVR) has emerged as an important tool in reducing stroke risk associated with this intervention. With the recent expansion of TAVR into lower-risk populations, the role of preventive strategies gained greater significance. Despite advancements in TAVR technologies, peri-procedural stroke remains a significant complication, with rates ranging between 2 and 5%. CEP devices, introduced at the time of the procedure, have been developed to capture embolic debris and reduce the risk of neurological events. However, while MRI-detected embolic debris is commonly captured by these devices, the clinical benefit in reducing stroke remains debated. This review provides a comprehensive analysis of recent advances in relevant clinical research and CEP device development, offering recommendations for future studies to improve patient outcomes.

## 1. Introduction

With population aging, the incidence of aortic stenosis (AS) has doubled in the last 20 years. This has led to higher rates of heart failure and mortality and has become a significant health and economic burden [1,2]. Transcatheter aortic valve replacement (TAVR) has revolutionized the treatment of severe aortic stenosis, especially in patients deemed too high-risk for conventional surgical aortic valve replacement. Irrespective of the presence of symptoms, the recommended treatment for severe aortic stenosis is heart valve replacement, either by TAVR or surgical intervention [3]. As technology has advanced, TAVR has become the preferred treatment for a broader range of patients, including those at moderate surgical risk. The increasing amount of evidence suggests TAVR could also become a viable and safe alternative for low-risk patients [4,5].

Despite the rapid development and evolution of TAVR devices and implantation techniques, which have shortened hospitalization and convalescence, the frequency of periprocedural complications remains high [6,7,8].

Cerebrovascular events are one of the most feared adverse events, significantly impacting quality of life, leading to disability and increased mortality. Peri-procedural stroke rates following TAVR are reported to range between 2 and 5%, with studies indicating even higher rates in specific subgroups, such as the elderly or those with heavily calcified aortic valves [9]. An analysis, conducted based on the STS/TVT registry, revealed the 30-day post-procedural stroke rate of 2.3%, resulting in 13% higher 30-day mortality and 43% lower hospital discharge rates than those without stroke [10].

The primary mechanism of stroke during TAVR involves embolic debris consisting of fragments of arterial wall, thrombi, valve tissue, and foreign materials that are liberated into the circulatory system displaced from the aortic wall during balloon valvuloplasty, valve positioning, or deployment, as was revealed in the dedicated histopathological analyses [11]. These emboli can cause both symptomatic strokes and silent brain infarcts, which are detectable in more than 70% of cases on diffusion-weighted MRI (DW-MRI) but often go unrecognized clinically. While silent infarcts may not cause immediate neurological deficits, evidence suggests that they may contribute to long-term cognitive decline, making embolic protection during TAVR an important consideration [12,13].

To address this risk, cerebral embolic protection (CEP) devices have been developed with the aim of capturing embolic debris before it reaches the cerebral vasculature (Table 1) [14]. These devices have been widely studied, with multiple clinical trials evaluating their efficacy [15]. While the devices appear effective in capturing embolic material, the clinical benefit in terms of stroke prevention remains less clear. This review provides a comprehensive overview of the current state of CEP devices in TAVR, focusing on the major clinical trials, and ongoing clinical development considerations, based on the available aggregated data from 2016 to the present.

## 2. Overview of Cerebral Embolic Protection Devices

### 2.1. Sentinel Cerebral Protection System

The Sentinel Cerebral Protection System (Boston Scientific) is the most widely studied CEP device and the only one currently approved for use in both Europe and the United States. The Sentinel device consists of two filters that are deployed in the brachiocephalic and left common carotid arteries via a right radial or brachial artery approach. These filters capture debris that would otherwise travel to the brain during TAVR. Several clinical trials have evaluated the efficacy of the Sentinel device in reducing stroke and neurological injury following TAVR.

The SENTINEL and CLEAN TAVI trials randomized TAVR patients to receive the Sentinel device or no protection. The studies demonstrated that the device successfully captured debris in over 99% of cases. However, while MRI studies showed a significant reduction in new brain lesions, there was no statistically significant reduction in overall stroke rates [11,16]. PROTECTED TAVR was a large, multicenter randomized study that included over 3000 patients and sought to determine whether the Sentinel device could reduce clinically significant stroke. While the overall stroke rate was not significantly different between the CEP and control groups (2.3% vs. 2.9%), there was a trend toward a reduction in disabling stroke in the CEP group (0.5% vs. 1.3%, *p* < 0.05). The number needed to treat (NNT) to prevent one disabling stroke was calculated to be 125 [17].

### 2.2. TriGuard 3 Cerebral Protection Device

The TriGuard 3 device (Venus Medtech Inc., Hangzhou, China) is a deflector-based system designed to protect all three major cerebral arteries (brachiocephalic, left common carotid, and left subclavian arteries) during TAVR. The device is deployed via the femoral artery and aims to provide broader protection compared to systems that only cover two of the three major vessels.

The DEFLECT III trial, one of the early studies assessing TriGuard’s efficacy, demonstrated a reduction in both clinical stroke and MRI-detected new brain lesions. However, this study was small and underpowered to detect differences in clinical stroke [18]. The REFLECT I and II trials further evaluated the efficacy and safety of TriGuard. While some benefit was seen in terms of lesion reduction, the impact on clinically evident stroke remained inconclusive [19,20].

### 2.3. ProtEmbo and Emboliner

Several other devices are currently in development or early clinical trials, including the ProtEmbo (Protembis) and Emboliner (Emboline, Inc.). The ProtEmbo device uses a heparin-coated mesh to capture debris, while Emboliner is designed for full-body embolic protection, capturing debris from both the cerebral and peripheral circulation. Early studies with these devices have shown promising results in terms of debris capture, but their impact on stroke prevention remains to be fully determined [21,22].

Both the ProtEmbo and Emboliner devices are currently being tested in their ongoing pivotal IDE studies—NCT05873816 and NCT05684146, respectively.

## 3. Predictors of Cerebrovascular Events and the Role of DW-MRI Lesions

Cerebrovascular events, particularly stroke and transient ischemic attack (TIA), are among the most concerning complications following transcatheter aortic valve replacement (TAVR). Predictors of such events can be broadly classified into those affecting early (acute and subacute) and late cerebrovascular events, and a range of both procedural and patient-related factors have been identified. Early events, typically occurring within the first 48 to 72 h post-TAVR, are heavily influenced by procedural complexities. For instance, patients with severe aortic valve calcification often undergo balloon post-dilatation, a procedure that has been strongly associated with an increased risk of early stroke [23]. The mechanical manipulation required during post-dilatation likely dislodges calcified material, leading to embolization. In a cohort study by Nombela-Franco et al., patients undergoing post-dilatation had significantly higher odds of developing early cerebrovascular events (OR, 2.46; 95% CI, 1.07–5.67), underscoring the impact of procedural factors on stroke risk [24].

On the other hand, the data from RCTs and national registries following the comparisons between balloon-expandable valves (BEVs) and self-expanding valves (SEVs) during TAVR revealed no significant differences in stroke risk. While some studies reported slight numerical variations in stroke risk between the two groups of valves (BEV and SEV) favoring BEV, due to factors such as the longer deployment time and additional manipulation required during SEV implantation, patient and procedural factors, including the need for post-dilatation, seem to play a more significant role than valve type alone [25,26,27].

Of note, the recently conducted LANDMARK trial compared early outcomes of a newer-generation SEV Myval transcatheter heart valve system with contemporary valves (Sapien and Evolut) and revealed a similarly low risk of stroke (3%) and disabling stroke (1%) in both groups [28].

Other procedural predictors of early stroke post-TAVR may include new-onset atrial fibrillation, which raises the likelihood of an ischemic stroke in the immediate post-procedural period [29]. Furthermore, rapid ventricular pacing (RVP), which reduces cardiac output, can cause transient hypotension and impact clinical outcomes. Although RVP is routinely performed during most TAVR procedures, current data on the clinical impact of procedural pacing only show a trend toward increased rates of stroke with longer durations of RVP [30]. In future, adequately designed and powered clinical studies are needed to confirm this correlation.

Interestingly, a recent study indicates that human aortic calcified valves constitute an important reservoir of thrombotic material, including procoagulant cell-derived extracellular vesicles (EVs). Within the native valve, EVs serve as potent biological agonists, transforming valvular endothelial cells and, in turn, the valve surface into a prothrombotic, proadhesive, and proinflammatory environment that attracts inflammatory cells and promotes thrombogenicity. Understanding the mechanisms behind prothrombotic activity in patients with aortic stenosis and following transcatheter aortic valve replacement is crucial and offers promising new therapeutic targets, such as those acting on AT1R/NADPH oxidases or SGLT2 pathways, to limit thromboembolic risk and curb valvular dysfunction [31].

The route of vascular access is another important procedural factor influencing stroke risk. Alternative access routes, such as transapical or transaortic approaches, have been associated with a higher risk of in-hospital stroke compared to the standard transfemoral approach [32]. A large-scale analysis of the TVT registry demonstrated that patients undergoing TAVR via non-transfemoral routes had the highest odds of in-hospital stroke, likely due to increased manipulation of the calcified aorta and the need for more invasive instrumentation [33]. This is particularly true for patients with heavily calcified or porcelain aortas, where the risk of embolization is significantly elevated. However, as operators gain more experience with alternative access routes, some studies have shown comparable stroke rates between transaortic and transfemoral approaches. Finally, it is worth noting that while CEP requires and utilizes one arterial access, which can restrict its applicability in patients where this access is limited, the alternative access route may provide a feasible option for those individuals.

Patient-specific factors also play a critical role in predicting early cerebrovascular events post-TAVR. Conditions such as chronic kidney disease, female sex, and a history of prior cerebrovascular events (e.g., stroke or TIA) have been consistently linked to a higher risk of stroke. Frailty, which encompasses a range of factors including low body mass, reduced mobility, and overall vulnerability, is also a key determinant of both early and late cerebrovascular events. In particular, frail patients with a history of falls or cognitive impairment are more susceptible to stroke due to the interplay between frailty, comorbid conditions, and procedural risks [30].

In contrast to early events, late cerebrovascular complications, which can occur weeks to months after TAVR, are more closely tied to a patient’s underlying cardiovascular health and comorbidities. Pre-existing atherosclerosis, atrial fibrillation, and ventricular dysfunction are among the most common predictors of late stroke. Persistent atrial fibrillation poses a significant thromboembolic risk, particularly when untreated or inadequately managed. Left ventricular dysfunction may contribute to poor cerebral perfusion, further increasing the risk of cerebrovascular events. Additionally, studies from trials such as PARTNER and CoreValve have identified prosthetic valve size as a significant predictor of late stroke. Patients receiving smaller prosthetic valves (e.g., 23 mm) were found to have higher rates of late cerebrovascular events, potentially due to residual hemodynamic stress or suboptimal valve function over time [34,35,36]. Finally, the cerebrovascular events risk may vary between surgical risk groups. Major TAVR vs. SAVR trials demonstrated 30-day risk rates which ranged from 3.8% to 5.0% [27,28,29]. In comparison, in low-risk TAVR populations trials, such as NOTION, PARTNER 3, and Evolut Low Risk, the 30-day stroke rates were 1.4%, 0%, and 0.5%, respectively [4,5,37]. Therefore, it is conceivable that patients with higher baseline risk would yield maximum therapy benefits over those with lower risks. The PROTECTED TAVR included patients at all levels of surgical risk, with an average STS score of 3.3% in the CEP arm and 3.4% in the control arm. While subgroup analyses of PROTECTED TAVR could not specify populations in whom CEP may have potential benefit, there was a signal towards absolute reduction in disabling stroke with CEP in patients with higher STS scores [17].

A significant recent development in understanding cerebrovascular risks post-TAVR is the role of diffusion-weighted MRI (DW-MRI) in detecting silent brain infarcts—small embolic lesions that do not result in immediate clinical symptoms but may contribute to long-term cognitive decline. DW-MRI has emerged as a key tool in identifying subclinical ischemic injury after TAVR, with multiple studies reporting that up to 85% of patients show signs of brain ischemia following the procedure [38]. While many of these lesions are not associated with overt neurological deficits, they are considered markers of embolic burden and potential contributors to late cognitive impairment. For example, a pooled analysis of data from trials including DEFLECT III and REFLECT I/II showed that patients with a total lesion volume (TLV) greater than 500 mm^3^ had a significantly higher risk of ischemic stroke and stroke-related disability compared to those with smaller lesion volumes. The presence of multiple lesions, even when subclinical, increases the cumulative risk of clinical stroke and other neurological events.

These MRI-detected lesions have significant clinical relevance, as evidenced by findings linking total lesion volume and individual lesion size to both early and late cerebrovascular events. Patients with higher lesion volumes were found to have a markedly elevated risk of stroke within 30 days of the procedure. For instance, patients with a TLV greater than 500 mm^3^ had an ischemic stroke rate of 18.2% compared to 2.3% in those with smaller lesion volumes, illustrating the prognostic importance of MRI findings. The incremental increase in stroke risk with larger lesion volumes suggests that DW-MRI may serve as a valuable surrogate marker for predicting future cerebrovascular complications and guiding the use of cerebral embolic protection devices (CEPDs).

The use of biomarkers to detect acute brain injury and predict cognitive dysfunction following cardiovascular interventions is an emerging area of research. Among the most studied biomarkers for brain injury related to these procedures are S100B, glial fibrillary acidic protein (GFAP), tau protein, neurofilament light polypeptide (NFL), and γ-enolase (neuron-specific enolase). S100B, a glia-derived protein, has shown particular promise, with elevated levels often correlating with neurological injury and cognitive decline after procedures like coronary artery bypass graft (CABG) surgery and transcatheter aortic valve replacement (TAVR). Studies have demonstrated that elevated S100B levels are associated with the detection of brain microemboli, as well as cognitive dysfunction post-intervention. Other biomarkers, such as GFAP, tau, and NFL, have also shown potential in detecting postoperative brain injury, although their predictive value remains modest. However, the nonspecific release of some of these biomarkers from extracerebral tissues, such as muscle and bone marrow, limits their diagnostic specificity [39]. Another study suggested that following TAVR, defects in high-molecular-weight multimers of von Willebrand factor (VWF), as assessed by closure time of adenosine diphosphate (CT-ADP) as a point-of-care measure of hemostasis, may contribute not only to bleeding but also to ischemic cerebrovascular events and may be considered potential predictors of early post-TAVR neurological incidents [40].

While no single biomarker has yet shown sufficient sensitivity and specificity to reliably detect ABI after cardiovascular procedures, ongoing research holds promise. Future cerebral embolic protection trials could benefit from incorporating these biomarkers to better understand and predict long-term neurological outcomes, as well as better stratify stroke risk and provide earlier intervention opportunities for patients with high embolic burden. The combination of procedural risk factors, such as valve calcification and post-dilatation, along with patient-specific predictors like atrial fibrillation and frailty, creates a multifactorial landscape for stroke risk post-TAVR. As research continues, the integration of DW-MRI findings and emerging biomarkers into stroke prediction models offers a promising avenue for improving patient outcomes and refining the use of embolic protection strategies. However, this approach has also its limitations. While DW-MRI is instrumental in detecting subclinical events, its correlation with long-term cognitive outcomes and functional disability remains debated. The reliance on imaging alone, without concurrent clinical assessments, limits our understanding of the true impact of silent infarcts.

## 4. Clinical Trial Considerations

The design of clinical trials for cerebral embolic protection devices (CEPDs) in TAVR presents several unique challenges. A key consideration, as is the case in most clinical trials, is the selection of appropriate endpoints. Traditionally, studies have focused on clinical stroke outcomes; however, as stroke is a relatively rare event, large sample sizes are required to demonstrate statistically significant differences. This has been a major limitation of many trials to date, which are often underpowered to detect small but meaningful differences in stroke rates. Consequently, the use of imaging-based surrogate endpoints has become increasingly relevant, especially given the low incidence of overt stroke in contemporary TAVR cohorts. Diffusion-weighted MRI (DW-MRI), which detects subclinical embolic lesions, provides a sensitive and objective measure of embolic burden during TAVR and has been widely used as a surrogate endpoint in CEPD trials. The challenge, however, lies in validating these imaging findings against clinically meaningful outcomes such as functional disability, cognitive decline, and long-term neurological deficits. Some researchers argue that while silent brain infarcts may be clinically benign in the short term, they represent cumulative damage to cerebral tissue, which over time could lead to cognitive deterioration. Others suggest that these MRI findings may not always predict functional decline, leading to questions about whether they should be prioritized as primary endpoints in trials [41].

A recent Bayesian meta-analysis conducted by Heuts et al. reviewed the available randomized controlled trials (RCTs) on CEP devices [42]. The study concluded that while there is a high probability that CEP devices reduce stroke, the reduction may not be clinically meaningful. The authors estimated a 44% relative risk reduction (RRR) in disabling stroke with CEP use, but the absolute risk reduction (ARR) was only 0.56%, leading to an NNT of 179. This finding highlights the difficulty in demonstrating large clinical benefits from CEP devices, despite their apparent efficacy in capturing embolic debris. Further, a newly conducted analysis of the STS/ACC TVT registry data, which evaluated a population consisting of >400,000 TAVR patients, of whom, 12.9% received an CEP, found that the use of an embolic protection device was associated with a reduction in risk of stroke associated with death or a discharge to a nonhome location (a selected proxy for disabling stroke), although the degree of benefit was small and of borderline statistical significance. While the results may be somewhat encouraging, they rather set the stage for future studies, as it remains unclear whether these data, using a proxy for stroke and showing only a modest decrease in events, are sufficient to change clinical practice [43].

Trials incorporating a composite or hierarchical endpoints, combining clinical stroke events with DW-MRI findings, may provide a more comprehensive assessment of CEPD efficacy. The inclusion of endpoints such as total lesion volume (TLV), individual lesion volume (ILV), and lesion count can help quantify the subclinical burden of embolization and allow for the more sensitive detection of differences between treatment groups. However, these surrogate endpoints must be validated through their correlation with long-term clinical outcomes, particularly the prevention of disabling strokes and cognitive impairment. This approach would enrich the understanding of the full spectrum of neurological outcomes post-TAVR.

The standardization of endpoints is also critical to ensure consistency across trials. The Valve Academic Research Consortium (VARC-3) guidelines provide a framework for defining and reporting neurological events in TAVR, including stroke, transient ischemic attacks (TIAs), and silent brain infarcts. These guidelines recommend standardized neurological assessments, ideally conducted by specialists, to ensure the accurate diagnosis and classification of cerebrovascular events. Adherence to VARC-3 criteria enhances comparability across studies and minimizes the risk of bias or misclassification.

A recent ongoing CEPD trial incorporated a composite endpoint of the Occurrence of Major Adverse Cardiac and Cerebrovascular Events (MACCE) at 30 days and TLV in the brain assessed by DW-MRI as a primary outcome measure. Interestingly, the trial aims to compare the safety and efficacy of the ProtEmbo Cerebral Embolic Protection device to a hybrid control—no embolic protection device and the industry benchmark Sentinel device. These head-to-head comparisons are equally important, and as newer CEP devices enter the market, future research should determine which designs offer the greatest benefit. Specifically, the efficacy of devices that protect all three major cerebral arteries, such as the ProtEmbo or TriGuard 3, should be evaluated against those that cover only two. Currently, both the ProtEmbo and Emboliner clinical trials are enrolling participants to demonstrate comparative data on efficacy and safety with regard to the Sentinel Cerebral Protection System.

Another important consideration is the sample size required to detect meaningful differences in stroke rates. Most conducted CEP trials were underpowered to detect statistically significant reductions in stroke rates, given the relatively low incidence of cerebrovascular events post-TAVR. While a large sample size of interventional device trials may require substantial financial investment, a focus on high-risk populations (e.g., patients with atrial fibrillation, prior cerebrovascular events, or heavily calcified aortic valves) may be necessary to ensure adequate statistical power. Trials that target these high-risk groups may observe higher event rates, making it easier to demonstrate the clinical efficacy of CEPD, leading to approval in populations with the highest unmet need. A recently published meta-analysis on cerebral embolic protection after TAVR (6 trials; n = 3921) stratified the trial results by baseline surgical risk and device type, suggesting that although the analysis did not demonstrate a statistically significant difference in any stroke between CEP and control groups, patients with high and intermediate surgical risks were most likely to derive benefits [41].

Follow-up duration is another critical aspect of trial design. Although most cerebrovascular events occur early after TAVR, and the CEP devices are removed shortly after the procedure, the potential for late stroke due to persistent embolic risk factors (such as atrial fibrillation) or prosthetic valve dysfunction persist. While it may not lead to a meaningful difference between tested groups, it may provide meaningful additional data to inform further device development strategies and designs. Cognitive decline, an important patient-centric measure, which may develop gradually over time, necessitates an extended follow-up period to fully capture the long-term impact of embolic events. The BHF PROTECT-TAVI trial, currently underway, has integrated cognitive testing and quality-of-life assessments into its follow-up protocol [44]. By utilizing tools such as the Montreal Cognitive Assessment (MoCA) and modified Rankin Scale (mRS), this trial aims to address a critical gap and provide a more comprehensive understanding of how embolic events, both silent and clinically apparent, affect long-term brain function and overall patient outcomes. Additionally, the inclusion of patient-reported outcomes (PROs) in future studies will be vital to gauge the real-world impact of these embolic protection strategies [45]. This can provide valuable insight into how patients experience their health post-procedure and whether CEP devices are translating into noticeable improvements in daily functioning, memory, and cognitive clarity. While improved survival following a cardiac intervention is crucial, patients value sustained physical function and the ability to engage in activities they enjoy. When deciding whether to participate in a cardiac clinical trial, they want to understand not only the short-term impact on their well-being post-surgery but also the long-term effects on physical function and social participation. Ultimately, patients prioritize feeling better, making it essential to measure PROs throughout the trial follow-up, particularly beyond the first year. Future CEP trials should routinely integrate PROs and extended cognitive testing, ensuring a more holistic evaluation of both short- and long-term impacts on patients’ health and quality of life. This aligns with contemporary research priorities, where patient-centered measures are becoming essential for assessing the full impact of interventions, particularly in an aging population. Including these metrics will better inform clinical decision-making and enhance the relevance of CEP device outcomes in real-world settings [45].

Finally, the cost-effectiveness of cerebral protection during TAVR is becoming an increasingly important factor for payers and health care systems, particularly as TAVR expands into lower-risk populations, and in greater numbers as the proportion of older people increases in the population. CEP devices, such as the Sentinel device, add procedural complexity and costs to TAVR. Although the devices themselves have been shown to be safe and effective at capturing embolic debris, the cost-effectiveness of routine CEP use remains debated. Since stroke increases the cost of the index hospitalization and doubles rehospitalization costs [46], incorporating economic analyses into trials, such as collecting data on resource utilization, can provide insights into whether the routine use of CEPDs justifies the additional procedural cost, particularly in terms of preventing strokes and reducing the long-term health care costs associated with post-stroke rehabilitation and care. While hospitalization rates in the long-term follow-up may provide beneficial data, it is unclear whether endpoints such as time to ICU or hospital discharge can bring significant insights into the overall effectiveness of the CEP intervention. This is due to a fact that the low cerebrovascular event rates may not influence this outcome significantly, and the variability in local discharge patterns after TAVR may further limit the potential for clear interpretation of these data points.

The BHF PROTECT-TAVI trial incorporated a cost-effectiveness model to estimate the short-term (1-year) and longer-term (lifetime) survival and quality-adjusted life years (QALYs) and the costs incurred by the NHS and Personal Social Services (PSS) to address whether value for money could be further optimized within the overall TAVR population using CEP (e.g., using additional risk stratification). The trial will prospectively link participants to collect further data on subsequent cerebrovascular events (from Hospital Episode Statistics, hospital resource utilization and mortality (Office for National Statistics)). Such linkages will provide a more complete picture of the short- and longer-term costs and outcomes and will enhance the precision and robustness of the cost-effectiveness results. Overall, tokenization—a concept of linking clinical trial participants to their real-world data—has become a viable and increasingly popular way to augment the understanding of patients’ health care experiences and the effects of treatments beyond clinical trials [47]. Linking RWD sources to trial data, while ensuring patients’ privacy, helps to address evidence gaps with outcome data and enables the generalization of randomized, controlled-derived data to real-world settings, and is highly encouraged.

By addressing the considerations of endpoint selection, standardization of outcomes, sample size, follow-up duration, and cost-effectiveness, future CEP devices trials can provide robust, clinically relevant, and economically viable results. These results will help guide the broader adoption of CEPDs in TAVR and ensure that the devices are used effectively to reduce stroke risk and improve long-term neurological outcomes in diverse patient populations.

Given the current landscape of TAVR procedures, clinicians should rely on a comprehensive assessment of patient-specific risk factors and procedural considerations to determine the appropriate use of cerebral embolic protection devices. CEPs are particularly beneficial for high-risk patients, such as those with advanced age, a history of cerebrovascular disease or previous stroke, atrial fibrillation, or a high burden of calcification in the aortic valve or arch. Anatomical considerations, like complex aortic anatomy or severe vascular tortuosity, also play a crucial role in the decision-making process. CT angiography is essential not only for determining the aortic annulus and access route but also for evaluating the anatomy of the aortic arch and supra-aortic vessels and recognizing specific anatomical features that are crucial for the successful deployment of CEP devices. Additionally, measuring the diameters of the supra-aortic vessels is necessary as, often, filters are designed to fit within a brachiocephalic trunk and a common carotid artery of a specified range. The use of fusion imaging, combining CTA-derived 3D anatomical models with live X-rays, can enhance deployment accuracy and minimize the need for contrast dye. Procedural factors, including the type of TAVR procedure and the devices used, can influence the decision, with balloon-expandable valves posing a higher risk of embolization compared to self-expanding valves. However, there are specific contraindications to consider, such as severe vascular tortuosity which precludes the safe introduction of the guiding catheter, known allergies or hypersensitivities to device materials or contrast media, uncorrected bleeding disorders, and technical limitations like the inability to cross the lesion with the filter delivery wire. The decision to use CEP devices should be individualized, balancing potential benefits against risks, and ideally made by a multidisciplinary heart team involving interventional cardiologists, neurologists, and imaging specialists. Future studies should aim to refine patient selection criteria and further elucidate the clinical benefits of CEP in diverse patient populations.

## 5. Conclusions

The use of cerebral embolic protection devices during transcatheter aortic valve replacement has shown promise in capturing embolic debris and reducing certain types of strokes, particularly disabling strokes. However, challenges remain in demonstrating their overall efficacy in reducing clinically significant stroke events and improving long-term cognitive outcomes. The results of recent meta-analyses and large-scale trials, such as PROTECTED TAVR, have tempered expectations about the routine use of CEP in TAVR, given the modest reductions in stroke rates.

## 6. Future Directions

The focus of future research should address the following gaps to offer clearer guidance on the role of CEP devices in reducing stroke, improving long-term outcomes, and thus providing clinically meaningful benefits for TAVR patients:

A. Expanded Trials: Larger, better-powered trials are needed to capture small but meaningful differences in stroke rates, especially in lower-risk populations. Alternatively, a focus on the most vulnerable populations with the highest unmet needs would provide meaningful clinical benefit.
B. Cognitive Function and Quality of Life: Extended follow-up with cognitive testing and patient-reported outcomes based on the linkage of trial participants with real-word data shoulpackad be a standard part of trial design.
C. Head-to-Head Device Comparisons: Comparative studies of different CEP designs will be crucial for determining which systems, designs, or structures offer the greatest protection.
D. Economic Evaluations: Cost-effectiveness analyses should be integrated into clinical trials to ensure that the benefits of CEP devices justify their use in routine practice.

## Figures and Tables

**Table 1:** Currently available cerebral embolic protection systems.

Device	SENTINEL™ Cerebral Protection System (Boston Scientific, Marlborough, MA, USA)	TriGuard 3™ (Venus Medtech Inc., Hangzhou, China)	ProtEmbo^®^ Cerebral Protection System (Protembis GmbH, Aachen, Germany)	Emblok™ Protection System (Innovative Cardiovascular Solutions, LLC, Grand Rapids, MI, USA)	Emboliner^®^ Total Embolic Protection Catheter (Emboline, Inc., Santa Cruz, CA, USA)	POINT-GUARD™ (Transverse Medical Inc., Denver, CO, USA)	CAPTIS^®^ Full-Body Embolic Protection Device (Filterlex Medical Ltd., Caesarea, Israel)	FLOWer (AorticLab, Colleretto Giacosa, Italy)
Coverage (targeted arteries)	Partial ^a^	Full Arch ^b^	Full Arch ^b^	Full Arch ^b^	Full Arch ^b^	Full Arch ^b^	Full Arch ^b^	Full Arch ^b^
Mechanism	Capture	Deflection	Deflection	Capture	Capture	Deflection	Capture	Capture
Mesh pore size	140 μm	115 × 145 μm	60 μm	125 μm	150 μm	105 μm	115 × 145 μm	70 μm
Vascular access	Right radial (6 Fr)	Femoral (8 Fr)	Left radial (6 Fr)	Femoral (11 Fr)	Femoral (10 Fr)	Femoral (10 Fr)	Femoral (16 Fr)	Femoral (12 Fr)

^a^—Two vessel protection (brachiocephalic and left common carotid artery). ^b^—Three vessel protection (brachiocephalic, left common carotid, and left subclavian artery). Fr—French.

## References

[1] Iung B., Delgado V., Rosenhek R., Price S., Prendergast B., Wendler O., De Bonis M., Tribouilloy C., Evangelista A., Bogachev-Prokophiev A. (2019). Contemporary presentation and management of valvular heart disease: The EURObservational Research Programme Valvular Heart Disease II Survey. Circulation.

[2] Roleder T., Hawranek M., Gąsior T., Cieśla D., Zembala M., Wojakowski W., Gąsior M., Gąsior Z. (2018). Trends in diagnosis and treatment of aortic stenosis in the years 2006–2016 according to the SILCARD registry. Pol. Arch. Intern. Med..

[3] Vahanian A., Beyersdorf F., Praz F., Milojevic M., Baldus S., Bauersachs J., Capodanno D., Conradi L., De Bonis M., De Paulis R. (2022). 2021 ESC/EACTS Guidelines for the management of valvular heart disease. Eur. Heart J..

[4] Mack M.J., Leon M.B., Thourani V.H., Pibarot P., Hahn R.T., Genereux P., Kodali S.K., Kapadia S.R., Cohen D.J., Pocock S.J. (2023). Transcatheter Aortic-Valve Replacement in Low-Risk Patients at Five Years. N. Engl. J. Med..

[5] Forrest J.K., Deeb G.M., Yakubov S.J., Gada H., Mumtaz M.A., Ramlawi B., Bajwa T., Teirstein P.S., Tchétché D., Huang J. (2023). 4-Year Outcomes of Patients With Aortic Stenosis in the Evolut Low Risk Trial. J. Am. Coll. Cardiol..

[6] Kim W.K., Brinkert M., Mangner N., Gatto F., Husser O., Renker M., Liebetrau C., Gasior T., Doss M., Walther T. (2019). Transfemoral implantation of the ACURATE neo prosthesis using a low-profile expandable introducer system: A multicenter registry. Int. J. Cardiol..

[7] Grodecki K., Tamarappoo B.K., Huczek Z., Jedrzejczyk S., Cadet S., Kwiecinski J., Rymuza B., Parma R., Olasinska-Wisniewska A., Fijalkowska J. (2021). Non-calcific aortic tissue quantified from computed tomography angiography improves diagnosis and prognostication of patients referred for transcatheter aortic valve implantation. Eur. Heart J. Cardiovasc. Imaging.

[8] Kochman J., Kołtowski Ł., Huczek Z., Rymuza B., Wilimski R., Dąbrowski M., Witkowski A., Grygier M., Olasińska-Wiśniewska A., Kubler P. (2018). Complete percutaneous approach versus surgical access in transfemoral transcatheter aortic valve implantation: Results from a multicentre registry. Kardiol. Pol..

[9] van Nieuwkerk A.C., Aarts H.M., Hemelrijk K.I., Urbano Carrillo C., Tchétché D., de Brito F.S., Barbanti M., Kornowski R., Latib A., D’Onofrio A. (2024). Cerebrovascular Events in Patients Undergoing Transfemoral Transcatheter Aortic Valve Implantation: A Pooled Patient-Level Study. J. Am. Heart Assoc..

[10] Huded C.P., Tuzcu E.M., Krishnaswamy A., Mick S.L., Kleiman N.S., Svensson L.G., Carroll J., Thourani V.H., Kirtane A.J., Manandhar P. (2019). Association Between Transcatheter Aortic Valve Replacement and Early Postprocedural Stroke. JAMA.

[11] Kapadia S.R., Kodali S., Makkar R., Mehran R., Lazar R.M., Zivadinov R., Dwyer M.G., Jilaihawi H., Virmani R., Anwaruddin S. (2017). Protection Against Cerebral Embolism During Transcatheter Aortic Valve Replacement. J. Am. Coll. Cardiol..

[12] Vermeer S.E., Prins N.D., den Heijer T., Hofman A., Koudstaal P.J., Breteler M.M. (2003). Silent brain infarcts and the risk of dementia and cognitive decline. N. Engl. J. Med..

[13] Sigurdsson S., Aspelund T., Kjartansson O., Gudmundsson E.F., Jonsdottir M.K., Eiriksdottir G., Jonsson P.V., van Buchem M.A., Gudnason V., Launer L.J. (2017). Incidence of Brain Infarcts, Cognitive Change, and Risk of Dementia in the General Population: The AGES-Reykjavik Study (Age Gene/Environment Susceptibility-Reykjavik Study). Stroke.

[14] Reddy P., Merdler I., Ben-Dor I., Satler L.F., Rogers T., Waksman R. (2024). Cerebrovascular events after transcatheter aortic valve implantation. EuroIntervention.

[15] Gasior T., Mangner N., Bijoch J., Wojakowski W. (2018). Cerebral embolic protection systems for transcatheter aortic valve replacement. J. Interv. Cardiol..

[16] Haussig S., Mangner N., Dwyer M.G., Lehmkuhl L., Lücke C., Woitek F., Holzhey D.M., Mohr F.W., Gutberlet M., Zivadinov R. (2016). Effect of a Cerebral Protection Device on Brain Lesions Following Transcatheter Aortic Valve Implantation in Patients with Severe Aortic Stenosis: The CLEAN-TAVI Randomized Clinical Trial. JAMA.

[17] Kapadia S.R., Makkar R., Leon M., Abdel-Wahab M., Waggoner T., Massberg S., Rottbauer W., Horr S., Sondergaard L., Karha J. (2022). Cerebral Embolic Protection during Transcatheter Aortic-Valve Replacement. N. Engl. J. Med..

[18] Lansky A.J., Schofer J., Tchetche D., Stella P., Pietras C.G., Parise H., Abrams K., Forrest J.K., Cleman M., Reinöhl J. (2015). A prospective randomized evaluation of the TriGuard™ HDH embolic DEFLECTion device during transcatheter aortic valve implantation: Results from the DEFLECT III trial. Eur. Heart J..

[19] Lansky A.J., Makkar R., Nazif T., Messé S., Forrest J., Sharma R., Schofer J., Linke A., Brown D., Dhoble A. (2021). A randomized evaluation of the TriGuard™ HDH cerebral embolic protection device to Reduce the Impact of Cerebral Embolic LEsions after TransCatheter Aortic Valve ImplanTation: The REFLECT I trial. Eur. Heart J..

[20] Nazif T.M., Moses J., Sharma R., Dhoble A., Rovin J., Brown D., Horwitz P., Makkar R., Stoler R., Forrest J. (2021). Randomized Evaluation of TriGuard 3 Cerebral Embolic Protection After Transcatheter Aortic Valve Replacement: REFLECT II. JACC Cardiovasc. Interv..

[21] Jagielak D., Targonski R., Frerker C., Abdel-Wahab M., Wilde J., Werner N., Lauterbach M., Leick J., Grygier M., Misterski M. (2022). Safety and performance of a novel cerebral embolic protection device for transcatheter aortic valve implantation: The PROTEMBO C Trial. EuroIntervention.

[22] Grubman D., Ahmad Y., Leipsic J.A., Blanke P., Pasupati S., Webster M., Nazif T.M., Parise H., Lansky A.J. (2023). Predictors of Cerebral Embolic Debris During Transcatheter Aortic Valve Replacement: The SafePass 2 First-in-Human Study. Am. J. Cardiol..

[23] Hahn R.T., Pibarot P., Leipsic J., Blanke P., Douglas P.S., Weissman N.J., Kapadia S., Thourani V.H., Herrmann H.C., Nazif T. (2018). The Effect of Post-Dilatation on Outcomes in the PARTNER 2 SAPIEN 3 Registry. JACC Cardiovasc. Interv..

[24] Nombela-Franco L., Rodés-Cabau J., DeLarochellière R., Larose E., Doyle D., Villeneuve J., Bergeron S., Bernier M., Amat-Santos I.J., Mok M. (2012). Predictive factors, efficacy, and safety of balloon post-dilation after transcatheter aortic valve implantation with a balloon-expandable valve. JACC Cardiovasc. Interv..

[25] Bhogal S., Waksman R., Shea C., Zhang C., Gordon P., Ehsan A., Wilson S.R., Levitt R., Parikh P., Bilfinger T. (2024). Self-expanding and balloon-expandable valves in low risk TAVR patients. Int. J. Cardiol..

[26] Deharo P., Bisson A., Herbert J., Lacour T., Saint Etienne C., Grammatico-Guillon L., Porto A., Collart F., Bourguignon T., Cuisset T. (2020). Impact of Sapien 3 Balloon-Expandable Versus Evolut R Self-Expandable Transcatheter Aortic Valve Implantation in Patients with Aortic Stenosis: Data From a Nationwide Analysis. Circulation.

[27] Lanz J., Kim W.K., Walther T., Burgdorf C., Möllmann H., Linke A., Redwood S., Thilo C., Hilker M., Joner M. (2019). Safety and efficacy of a self-expanding versus a balloon-expandable bioprosthesis for transcatheter aortic valve replacement in patients with symptomatic severe aortic stenosis: A randomised non-inferiority trial. Lancet.

[28] Baumbach A., van Royen N., Amat-Santos I.J., Hudec M., Bunc M., Ijsselmuiden A., Laanmets P., Unic D., Merkely B., Hermanides R.S. (2024). LANDMARK comparison of early outcomes of newer-generation Myval transcatheter heart valve series with contemporary valves (Sapien and Evolut) in real-world individuals with severe symptomatic native aortic stenosis: A randomised non-inferiority trial. Lancet.

[29] Ryan T., Grindal A., Jinah R., Um K.J., Vadakken M.E., Pandey A., Jaffer I.H., Healey J.S., Belley-Coté É.P., McIntyre W.F. (2022). New-Onset Atrial Fibrillation After Transcatheter Aortic Valve Replacement: A Systematic Review and Meta-Analysis. JACC Cardiovasc. Interv..

[30] Fefer P., Bogdan A., Grossman Y., Berkovitch A., Brodov Y., Kuperstein R., Segev A., Guetta V., Barbash I.M. (2018). Impact of Rapid Ventricular Pacing on Outcome After Transcatheter Aortic Valve Replacement. J. Am. Heart Assoc..

[31] Hmadeh S., Trimaille A., Matsushita K., Marchandot B., Carmona A., Zobairi F., Sato C., Kindo M., Hoang T.M., Toti F. (2024). Human Aortic Stenotic Valve-Derived Extracellular Vesicles Induce Endothelial Dysfunction and Thrombogenicity Through AT1R/NADPH Oxidases/SGLT2 Pro-Oxidant Pathway. JACC Basic Transl. Sci..

[32] Kapadia S., Agarwal S., Miller D.C., Webb J.G., Mack M., Ellis S., Herrmann H.C., Pichard A.D., Tuzcu E.M., Svensson L.G. (2016). Insights into Timing, Risk Factors, and Outcomes of Stroke and Transient Ischemic Attack After Transcatheter Aortic Valve Replacement in the PARTNER Trial (Placement of Aortic Transcatheter Valves). Circ. Cardiovasc. Interv..

[33] Arnold S.V., Manandhar P., Vemulapalli S., Vekstein A.M., Kosinski A.S., Carroll J.D., Thourani V.H., Mack M.J., Cohen D.J. (2023). Mediators of Improvement in TAVR Outcomes Over Time: Insights From the STS-ACC TVT Registry. Circ. Cardiovasc. Interv..

[34] Smith C.R., Leon M.B., Mack M.J., Miller D.C., Moses J.W., Svensson L.G., Tuzcu E.M., Webb J.G., Fontana G.P., Makkar R.R. (2011). Transcatheter versus surgical aortic-valve replacement in high-risk patients. N. Engl. J. Med..

[35] Leon M.B., Smith C.R., Mack M., Miller D.C., Moses J.W., Svensson L.G., Tuzcu E.M., Webb J.G., Fontana G.P., Makkar R.R. (2010). Transcatheter aortic-valve implantation for aortic stenosis in patients who cannot undergo surgery. N. Engl. J. Med..

[36] Popma J.J., Adams D.H., Reardon M.J., Yakubov S.J., Kleiman N.S., Heimansohn D., Hermiller J., Hughes G.C., Harrison J.K., Coselli J. (2014). Transcatheter aortic valve replacement using a self-expanding bioprosthesis in patients with severe aortic stenosis at extreme risk for surgery. J. Am. Coll. Cardiol..

[37] Thyregod H.G., Steinbrüchel D.A., Ihlemann N., Nissen H., Kjeldsen B.J., Petursson P., Chang Y., Franzen O.W., Engstrøm T., Clemmensen P. (2015). Transcatheter Versus Surgical Aortic Valve Replacement in Patients with Severe Aortic Valve Stenosis: 1-Year Results from the All-Comers NOTION Randomized Clinical Trial. J. Am. Coll. Cardiol..

[38] Lansky A.J., Grubman D., Dwyer M.G., Zivadinov R., Parise H., Moses J.W., Shah T., Pietras C., Tirziu D., Gambone L. (2024). Clinical Significance of Diffusion-Weighted Brain MRI Lesions After TAVR: Results of a Patient-Level Pooled Analysis. J. Am. Coll. Cardiol..

[39] Lenarczyk R., Proietti M., Scheitz J.F., Shah D., Siebert E., Gorog D.A., Kowalczyk J., Bonaros N., Ntaios G., Doehner W. (2024). Clinical and subclinical acute brain injury caused by invasive cardiovascular procedures. Nat. Rev. Cardiol..

[40] Matsushita K., Marchandot B., Trimaille A., Kibler M., Heger J., Peillex M., Hess S., Grunebaum L., Reydel A., Kindo M. (2020). Paradoxical Increase of Stroke in Patients with Defect of High Molecular Weight Multimers of the von Willebrand Factors following Transcatheter Aortic Valve Replacement. Thromb. Haemost..

[41] Khan S.U., Zahid S., Alkhouli M.A., Akbar U.A., Zaid S., Arshad H.B., Little S.H., Reardon M.J., Kleiman N.S., Goel S.S. (2023). An Updated Meta-Analysis on Cerebral Embolic Protection in Patients Undergoing Transcatheter Aortic Valve Intervention Stratified by Baseline Surgical Risk and Device Type. Struct. Heart..

[42] Heuts S., Gabrio A., Veenstra L., Maesen B., Kats S., Maessen J.G., Walton A.S., Nanayakkara S., Lansky A.J., van ‘t Hof A.W.J. (2024). Stroke reduction by cerebral embolic protection devices in transcatheter aortic valve implantation: A systematic review and Bayesian meta-analysis. Heart.

[43] Butala N.M., Kapadia S.R., Secemsky E.A., Gallup D., Kosinski A.S., Vemulapalli S., Messenger J.C., Yeh R.W., Cohen D.J. (2024). Impact of Cerebral Embolic Protection Devices on Disabling Stroke After TAVR: Updated Results From the STS/ACC TVT Registry. Circ. Cardiovasc. Interv..

[44] Kharbanda R.K., Perkins A.D., Kennedy J., Banning A.P., Baumbach A., Blackman D.J., Dodd M., Evans R., Hildick-Smith D., Jamal Z. (2023). Routine cerebral embolic protection in transcatheter aortic valve implantation: Rationale and design of the randomised British Heart Foundation PROTECT-TAVI trial. EuroIntervention.

[45] Masterson Creber R., Spadaccio C., Dimagli A., Myers A., Taylor B., Fremes S. (2021). Patient-Reported Outcomes in Cardiovascular Trials. Can. J. Cardiol..

[46] Alqahtani F., Sengupta P.P., Badhwar V., McCarthy P., Alkhouli M. (2019). Clinical and Economic Burden of Acute Ischemic Stroke Following Transcatheter Aortic Valve Replacement. Struct. Heart.

[47] Eckrote M.J., Nielson C.M., Lu M., Alexander T., Gupta R.S., Low K.W., Zhang Z., Eliazar A., Klesh R., Kress A. (2024). Linking clinical trial participants to their U.S. real-world data through tokenization: A practical guide. Contemp. Clin. Trials Commun..

